# Anti-Pneumococcal Properties of the Native Human Milk Oligosaccharide Fraction: A Concentration-Dependent Study

**DOI:** 10.3390/ijms262110781

**Published:** 2025-11-06

**Authors:** Oliwia Makarewicz, Tinatini Tchatchiashvili, Lisa Jasef, Mark P. G. van der Linden, Sylwia Jarzynka, Kamila Strom, Nico Ueberschaar, Maciej Mazur, Gabriela Oledzka, Mathias W. Pletz

**Affiliations:** 1 Institute of Infectious Diseases and Infection Control, Jena University Hospital, Friedrich Schiller University Jena, Am Klinikum 1, 07747 Jena, Germany; tinatini.tchatchiashvili@med.uni-jena.de (T.T.); lisa.jasef@med.uni-jena.de (L.J.); mathias.pletz@med.uni-jena.de (M.W.P.); 2 Uniklinik RWTH Aachen, Institute of Medical Microbiology, Referenzlabor für Streptokokken, Pauwelstraße 30, 52074 Aachen, Germany; mlinden@ukaachen.de; 3 Department of Medical Biology, Medical University of Warsaw, Litewska 14/16, 00-575 Warsaw, Poland; sylwia.jarzynka@wum.edu.pl (S.J.); kamila.strom@wum.edu.pl (K.S.); gabriela.oledzka@wum.edu.pl (G.O.); 4 Mass Spectrometry Platform, Friedrich Schiller University Jena, Humboldtstraße 8, 07743 Jena, Germany; nico.ueberschaar@uni-jena.de; 5 Faculty of Chemistry, University of Warsaw, Pasteura 1, 02-093 Warsaw, Poland; mmazur@chem.uw.edu.pl

**Keywords:** adjuncts, antimicrobials, bacteria, kinetic, pneumonia, vaccine

## Abstract

*Streptococcus pneumoniae* is a major opportunistic pathogen and a leading cause of severe infections in infants under two years of age. Human milk oligosaccharides (HMOs), key bioactive components of breast milk, possess immunomodulatory and antimicrobial properties. In this study, the antipneumococcal effects of HMOs are investigated across multiple *S. pneumoniae* serotypes, focusing on concentration-dependent activity and underlying mechanisms. Growth inhibition and bacterial viability were evaluated using growth curve analysis and colony-forming unit (CFU) assays. HMOs inhibited pneumococcal growth in a concentration-dependent manner, with suppression observed at 1.5–2.5 mg/mL and complete killing at 5 mg/mL for all serotypes. Nonencapsulated strains were more sensitive, with inhibition at 1 mg/mL. In the CFU assays, killing occurred at 1.25–5 mg/mL depending on the strain. At physiologically relevant colostrum concentrations (20–25 mg/mL), HMOs achieved complete bactericidal effects across all the tested strains. In contrast, lactose at equivalent doses showed no measurable antimicrobial activity, confirming the specificity of the observed effects. Overall, HMOs exhibit serotype-independent antipneumococcal activity, possibly through interference with bacterial adhesion or metabolic disruption. These findings suggest a potential role for HMOs as adjunctive agents in the prevention of pneumococcal infections in vulnerable populations, such as infants, and warrant further in vivo studies to validate these effects and explore clinical applications.

## 1. Introduction

*Streptococcus pneumoniae*, commonly known as pneumococcus, is a Gram-positive bacterium that frequently colonizes the human nasopharynx and poses a significant public health threat, particularly to infants and elderly individuals. Pneumococci can cause severe, life-threatening infections, including pneumonia, bloodstream infections, and meningitis. When the bacteria invade sterile sites in the body, these infections are classified as invasive pneumococcal disease (IPD). Pneumococci are also a leading cause of noninvasive infections such as otitis media [1]. These infections contribute to substantial morbidity and mortality worldwide, particularly in young children in developing countries where vaccination coverage may be incomplete. The risk of infection in elderly individuals is increased by close contact with colonized or infected children, who are the primary reservoir and often asymptomatic carriers of pneumococci. Compared with adults, children have substantially higher rates of nasopharyngeal colonization, which can lead to increased transmission within households and community settings, posing a significant risk to older adults with weakened immune systems or comorbidities [2,3]. Conversely, compared with grandparents or caregivers, the parental generation (typically middle-aged adults) tends to have lower infection risks because of a combination of acquired immunity from previous exposure and a lower likelihood of close, frequent contact with colonized young children. The primary pneumococcal virulence factor essential for immune evasion is the polysaccharide capsule, whose molecular composition defines a specific serotype. To date, more than 100 pneumococcal serotypes have been identified; however, only a subset is responsible for the majority of IPD [4].

Although vaccination targeting the pneumococcal capsule is crucial for controlling pneumococcal disease, not all vaccines confer equal protection across age groups. For instance, the 23-valent pneumococcal polysaccharide vaccine targets 23 pneumococcal serotypes and is primarily used in adults because of its notably low immunogenicity in infants under two years of age. At this early developmental stage, the immature immune system often fails to mount an optimal response to polysaccharide antigens. Consequently, pneumococcal conjugate vaccines (PCVs) are preferred in pediatric immunization programs. Unlike pure polysaccharide vaccines, conjugate vaccines elicit a stronger and longer-lasting immune response by engaging helper T cells, thereby providing enhanced protection against pneumococcal infections in young children.

PCVs have been shown to be suitable for vaccinating children under five years of age, significantly reducing the incidence of IPD not only among vaccinated children but also among unvaccinated individuals through herd immunity [5,6]. PCV7, introduced in 2000 by Wyeth Pharmaceuticals (now part of Pfizer), targeted seven serotypes (4, 6B, 9V, 14, 18C, 19F, and 23F) responsible for most invasive pneumococcal diseases at the time. PCV7 was followed by the development of PCV13 in 2010, which expanded coverage to include six additional serotypes (1, 3, 5, 6A, 7F, and 19A). PCV10 (Synflorix), which offers protection against ten serotypes, has been widely used in various immunization programs, particularly in Europe and other regions. More recently, PCV15 (Vaxneuvance), which includes serotypes 1, 3, 5, 6A, 7F, 19A, 22F and 33F, has been introduced as an alternative to PCV13. In June 2021 and February 2022, the PCV20 vaccine (Prevnar 20, covering serotypes 1, 3, 4, 5, 6A, 6B, 7F, 8, 9V, 10A, 11A, 12F, 14, 15B, 18C, 19A, 19F, 22F, 23F, and 33F) was approved for adult immunization in the United States and Europe. However, serotype replacement has emerged as a concern, with nonvaccine serotypes increasing in prevalence, causing IPD, and abolishing the benefit of vaccination in several regions [7]. Moreover, recent studies indicate that nonencapsulated *S. pneumoniae* (NESp) represents an underestimated risk for pneumococcal infections, as these strains are not targeted by current polysaccharide-based vaccines. Its prevalence among human-carried isolates ranges from 4% to 19% worldwide, with higher rates observed in vaccinated populations [8]. Compounding these challenges, certain vaccine-targeted serotypes—most notably serotype 3 (ST3)—exhibit diminished vaccine-induced immune responses, thereby complicating efforts to control IPD [9]. The replacement of emergence serotypes underscores the need for novel preventive and therapeutic strategies that are particularly effective against vaccine-resistant strains such as nonvaccine serotypes and NESp.

Breastfeeding has long been associated with numerous health benefits for infants, including a reduced frequency of severe infections. This protective effect is partly attributed to the presence of human milk oligosaccharides (HMOs) in breast milk that exceed the concentration of oligosaccharides in bovine milk by 100 to 1000-fold [10]. These complex carbohydrates constitute the third largest fraction after lactose and fats in human milk and play a critical role in infant health, not only by shaping the gut microbiome but also through their prebiotic, immunomodulatory, and antimicrobial activities [11,12,13]. They have also been detected in the systemic circulation of infants [14], suggesting that they may reach various organs, including the lungs and brain. The bioactivity of HMOs is largely determined by their structural diversity, with more than 200 distinct HMO structures identified thus far. In general, the backbone of HMOs is composed of 3 to 15 monosaccharide units of galactose, glucose, and N-acetyl-glucosamine that can be linked via β1-4, β1-3 or β1-6 glycosidic bonds, forming linear and branched chains. These can be further fucosylated, acetylated or sialylated [15]. This diversity allows for HMOs to perform multiple biological functions, including immune modulation and antimicrobial activity [16,17].

As previously hypothesized and partially demonstrated through individual in vitro studies, the antimicrobial (and antiviral) effects of HMOs likely arise from inhibiting or preventing pathogens from binding to their specific cellular receptors. As small soluble compounds, HMOs pass through the intestinal barrier and can bind to pathogens or host cells via carbohydrate–carbohydrate interactions, blocking these receptors [18,19].

Studies have demonstrated that HMOs can inhibit the growth of pathogenic bacteria, including Gram-positive species such as *Staphylococcus aureus* [20] and *Streptococcus agalactiae* [21,22]. Therefore, we aimed to investigate the antipneumococcal activity of HMOs. We hypothesized that HMOs might generally protect the nasopharynx of breastfed infants from infections by *S. pneumoniae*, a major human pathogen, particularly for this age group. As the *S. pneumoniae* serotype may play a role in the susceptibility to HMOs, we tested the effectiveness of HMOs against four common pneumococcal serotypes, including ST3, and several NESp isolates.

## 2. Results

### 2.1. Analysis of the HMO Fraction

Analysis of the total HMO fraction using extracted ion chromatogram (EIC) integration revealed that the preparation was predominantly composed of disaccharides, which accounted for approximately 77.7% of the total saccharide signal intensity (Table S1). Trisaccharides represented the second most abundant group (21.2%), while higher oligosaccharides (tetra- to hexasaccharides) collectively contributed less than 1% of the total signal. Monosaccharides were detected only in trace amounts (0.14%). These results confirm that the analyzed fraction was largely dominated by disaccharides, which is consistent with the high lactose content expected for native human milk oligosaccharide extracts.

**Figure 1:**
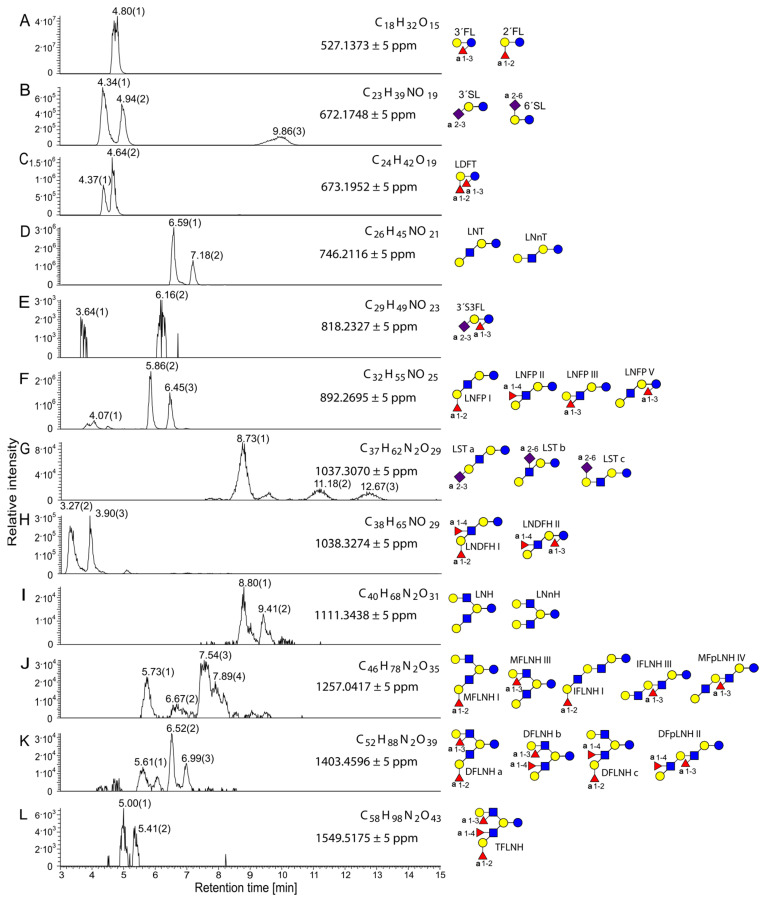
Extracted ion chromatograms of the [M+K]^+^ ions of HMOs from undigested sample 3, showing molecular formulas, exact masses, and possible structural assignments. The relative intensity is plotted against retention time. Measurements were performed in positive ionization mode using the Hypercarb column. (**A**) 2′FL (2′-fructosyllactose) and 3′FL (3′-fructosyllactose), (**B**) 3′SL (3′-sialyllactose) and 6′SL (6′-sialyllactose), (**C**) LDFT (lactodifructotetraose), (**D**) LNT F(lacto-*N*-tetraose), LNnT (lacto-*N*-neotetraose), (**E**) 3′S3FL (3′-sialyl-3-fucosyllactose), (**F**) LNFP I, II, III, and V (lacto-*N*-fucopentaose I, II, III, and V), (**G**) LSTa, b, and c (lacto-*N*-sialyltetraose a, b, and c), (**H**) LNDFH I and II (lacto-*N*-difucohexaose I and II), (**I**) LNH (lacto-*N*-hexaose), LNnH (lacto-*N*-neohexaose), (**J**) MFLNH I and III (monofucosyllacto-*N*-hexaose I and III), IFLNH I and III (isomerfucosyllacto-*N*-hexaose I and III), MFpLNH IV (monofucosyl-para-lacto-*N*-hexaose IV), (**K**) DFLNHa, b, and c (difucosyllacto-*N*-hexaose a, b, and c), DFpLNH II (difucosyl-para-lacto-*N*-hexaose II), (**L**) TFLNH (trifucosyllacto-*N*-hexaose). Referring to the Symbol Nomenclature for Glycans: yellow circle, galactose; blue circle, glucose; blue square, *N*-acetylglucosamine; red triangle, fucose; purple diamond, sialic acid.

Chromatographic analysis using a porous graphitic-carbon (Hypercarb) column provided clear separation of major HMOs, revealing characteristic retention patterns for neutral and acidic species [23,24]. Orbitrap high-resolution mass spectrometry (HRMS) enabled the identification of principal neutral and sialylated HMOs and the observed mass-spectral fingerprints and relative proportions, with the highest abundance of 2′-fucosyllactose (2′-FL) and 3′-fucosyllactose (3′-FL), lacto-di-fructotetraose (LDFT), lacto-*N*-tetraose (LNT), lacto-*N*-neotetraose (LNnT), lacto-*N*-fucopentaose I–III (LNFP I–III), and lacto-*N*-di-fucohexaose I/II (LNDFH I/II), followed by sialylated structures 3′-sialyllactose (3′-SL) and 6′-sialyllactose (6′-SL) (Figure 1).

To determine the chemical purity of the HMO fraction, we performed 3 different complementary methods, namely, proton NMR and two LC–MS analyses (C18 and ZIC-HILIC approaches).

The ^1^H-NMR spectrum (Figure S1) displayed a dense cluster of signals between 1.0 and 5.5 ppm, typical for carbohydrate protons, corresponding to an estimated 60.1 relative protons assignable to glycosidic structures [25]. Two minor doublets between 6 and 7 ppm (2.2 relative protons) could not be attributed to saccharide protons and likely indicate trace nonglycosidic contaminants.

C18 chromatography focuses on mid-polar to unpolar molecules, while ZIC-HILIC separation focuses on polar compounds. Metabolomics relies on in silico processing of data obtained from the LC–MS instrument. We therefore filtered the data according to their peak shape with a peak rating ≥ 6 and a retention time > 0.9 min since compounds elute earlier or elute with the column dead volume and thus cannot be compared. Additionally, we ran a solvent blank and set a filter to sort out compounds that were less than 2-fold more abundant in the HMO fraction than in the solvent blank. To focus on the most abundant compounds, only hits > 5 × 10^6^ for C18 are shown in Table S2. Since HILIC has a focus on polar compounds and thus ionizable compounds can be detected better, we set the threshold to >5 × 10^7^. (Note that under these conditions, the sugars do not ionize well; thus, they are underrepresented. Only the HILIC chromatography results show sugar-related compounds (Table S3).

LC–MS profiling using C18 and ZIC-HILIC separations revealed, in addition to the dominant sugar-derived ions, several low-abundance fatty acid derivatives, phospholipid fragments, and peptide-like residues (Tables S2 and S3). Notably, compounds such as oleic acid, palmitic acid, arachidonic acid, and glycerophosphocholine derivatives were still detectable despite prior defatting and protein precipitation of the milk prior to HMO purification. Their presence indicates incomplete removal of lipid-associated molecules that may coelute with complex oligosaccharides during extraction. However, the proton-NMR spectrum revealed at least 95% purity of the HMOs comparing to the discussed minor abundant impurities.

### 2.2. Effects of HMOs on Growth Curves

In general, all the tested *S. pneumoniae* isolates exhibited relatively weak growth under laboratory conditions, reaching a maximum optical density at 600 nm (OD_600_) below 0.25 within approximately 6 h. Notably, after the maximum was reached, the OD_600_ decreased below the initial inoculum level for most isolates during the death phase, indicating cell lysis (Supplementary Materials, Figures S2–S7). Compared with the encapsulated isolates, the NESp isolates generally tended to grow more weakly under these conditions.

Lactose had no significant effect on the growth curves of any of the isolates. Occasionally, slightly higher lactose concentrations (above 4 mg/mL) resulted in minor growth impairments, reflected by a slightly lower maximum achievable optical density.

The HMOs clearly reduced the growth of all the isolates tested in a concentration-dependent manner. Within the encapsulated serotypes, the effect of HMOs on growth was particularly pronounced in the ST6B isolates, with one isolate already showing inhibition at 1 mg/mL and the other at concentrations above 1.5 mg/mL and 2 mg/mL HMOs. In ST3, growth was inhibited at HMO concentrations above 2 mg/mL. The ST14 isolates seemed somewhat less sensitive, with growth inhibition observed only at concentrations above 2.5 mg/mL, similar to two of the ST19A isolates. Only one of the ST19A isolates showed growth inhibition starting from 2 mg/mL HMOs.

The NESp isolates appeared to be more sensitive to HMOs, with four isolates being fully inhibited at the lowest HMO concentration used (1 mg/mL). The growth of the other three isolates was visibly impaired by 2 mg/mL HMOs.

The significant reduction in OD_600_, which decreased below zero after normalization, strongly implied that bacterial cell death occurred at higher HMO concentrations, most likely releasing enzymes that degraded broth compounds, resulting in clearance of the medium.

### 2.3. Quantitative Parameters to Describe the Effect of HMOs on Pneumococcal Growth

To quantify the effects of HMOs on pneumococci, we extracted useful parameters from the normalized growth curves: the maximal OD_600_ (OD_MAX_), which corresponds to the number of bacteria grown under the respective conditions; the time to achieve the OD_MAX_ (t_MAX_), which reflects delays in growth caused by the HMOs; and the maximum achieved growth rate (µ_MAX_), which is determined at the curve inflection point and indicates the transition from exponential to inhibited growth and can be derived from the first derivative of the curve.

**Figure 2:**
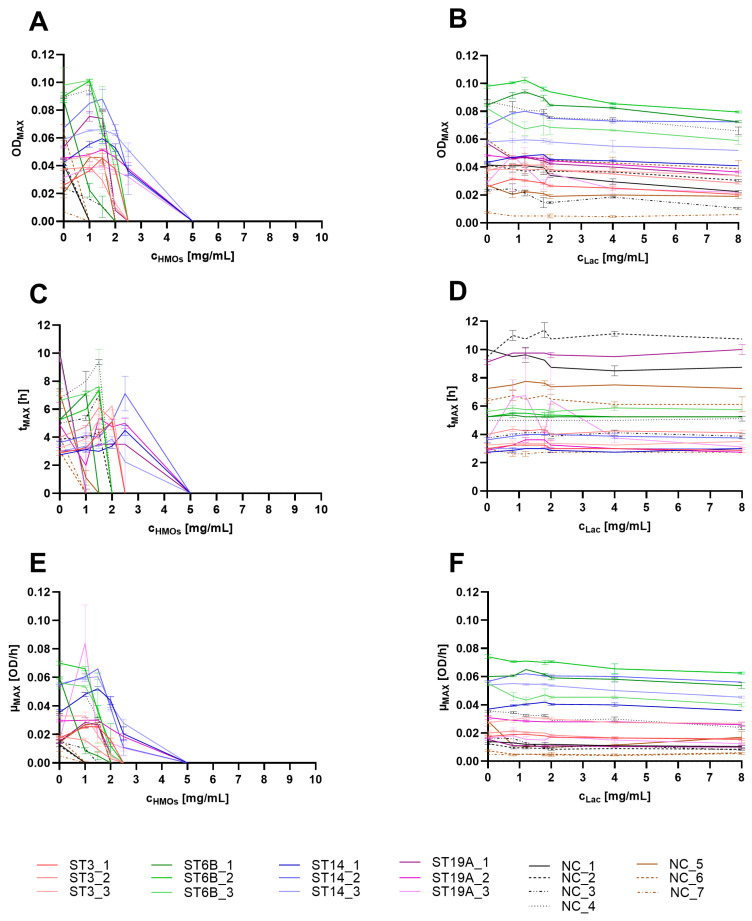
Growth parameters OD_MAX_ (**A**,**B**), t_MAX_ (**C**,**D**) and µ_MAX_ (**E**,**F**) extracted from the growth curves of different *S. pneumoniae* strains treated with HMOs (**A**,**C**,**E**) or equivalent lactose (**B**,**D**,**F**) concentrations. The strains are indicated in the legend. The experiments were performed in duplicate; the mean and range are shown in the graphs. In the legend, ST refers to the serotype, and NC indicates nonencapsulated (NESp isolates).

In most encapsulated pneumococci, the OD_MAX_ first increased slightly to a maximum of 1.5 mg/mL HMOs but decreased thereafter with increasing HMO concentration (Figure 2A). The t_MAX_ was proportional to the OD_MAX_ and increased in a HMO concentration-dependent manner if any OD_MAX_ could be determined (Figure 2C). With HMO concentrations above 2.5 mg/mL, the OD_MAX_ equaled the inoculum and was therefore set to ‘zero’. Consequently, no t_MAX_ could be determined, as HMOs reduced bacterial density immediately after the start of incubation, potentially through bactericidal effects. The µ_MAX_ values were similar (Figure 2E); if growth could be observed at lower HMO concentrations (to a maximum of 1.5 mg/mL), the µ_MAX_ increased slightly compared with that of the untreated control and decreased strongly with higher HMO concentrations, reaching ‘zero’ above 2.5 mg/mL at growth inhibition and even lysis.

Isolate-specific differences in growth were evident, but after the growth parameters were compared, it was not possible to conclusively determine whether there were significant serotype-specific differences. The differences were too high within the same serotypes, with most of the variance occurring within the ST19A and NESp strains. 8There were also no clear distinctions in the growth parameters between the encapsulated and NESp isolates. However, the observed effects on the OD_MAX,_ t_MAX_, and µ_MAX_ were more pronounced in NESp isolates, suggesting that the presence of the capsule might have a weak but protective effect on the mode of action of the HMOs.

Lactose did not affect the growth of any of the tested strains; thus, none of the three parameters differed between treated and untreated cultures, even at the highest lactose concentrations (Figure 2B,D,F).

The results suggest that at low HMO concentrations (below 1 mg/mL), the growth of the capsulated serotypes may not be significantly impaired, even though the cultures reached the OD_MAX_ somewhat later than the untreated control did. Conversely, concentrations above 2.5 mg/mL resulted in visible growth inhibition.

### 2.4. Viable Bacteria Count in Comparison to the Growth Parameters

**Figure 3:**
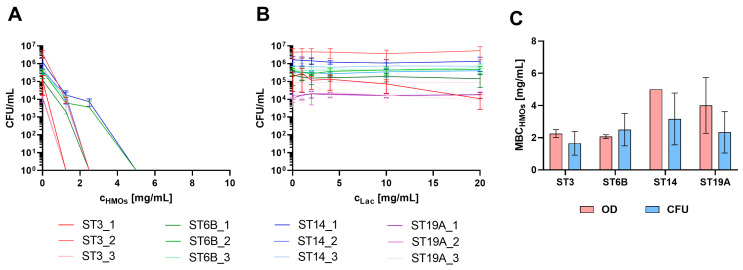
Quantification of CFU/mL following treatment with HMOs (**A**) or equivalent lactose concentrations (**B**). In Panel (**A**), the CFU/mL was set to 1 for the first concentration at which no growth was observed to indicate the killing concentration (as values below 0 cannot be represented on a logarithmic scale), and the concentration range shown extends to 10 mg/mL for visualization purposes. The legends for (**A**,**B**) are below the diagrams. (**C**) Minimal bactericidal concentration of the HMOs (MBC_HMOs_), assessed as the first nongrowth concentration on the basis of CFU or OD_600_ data of the vaccine serotypes.

To confirm the bactericidal effect of HMOs on pneumococci, we quantified the number of colony-forming units per millilitre (CFU/mL) of encapsulated *S. pneumoniae* isolates after a 6 h growth period in TM medium (Figure 3). The tested concentrations were slightly modified to keep the number of plates manageable while also increasing the maximum concentration (to 25 mg/mL HMOs and 20 mg/mL lactose) to ensure that no unexpected growth occurred on the plates. This adjustment was necessary since growth curves represent an indirect measure and do not directly reflect viability.

For some strains, no CFUs could be detected at concentrations as low as 1.25 mg/mL, whereas for others, this was observed only at higher concentrations of 2.5 mg/mL or 5 mg/mL. Above these concentrations, no growth was detected, indicating the strong efficacy of HMOs against pneumococci at the concentrations expected in breast milk.

A correlation between the bactericidal concentration and a specific serotype was not observed, suggesting that the efficiency of HMOs was not dependent on the capsular composition but rather on other isolate-specific factors that remained unidentified. NESp isolates were not included in this experiment, as they already demonstrated significantly higher sensitivity to HMOs in growth curve assays. Therefore, we assumed that the killing concentrations for NESp isolates would also fall below 5 mg/mL.

On the basis of the results, the most indicative parameter of HMO killing ability appears to be the number of CFUs/mL, as it directly reflects bacterial viability. This parameter provides a definitive assessment of whether pneumococci are viable after exposure to HMOs and highlights the bactericidal effect. However, as the growth of pneumococci is generally weak, one might overestimate the effect when samples are collected after the growth passes the maximum and lysis begins. When the minimal bactericidal concentration (MBC) values (first nongrowth concentration) determined from the CFU and OD measurements were compared, both methods yielded comparable results for the respective strains. However, the variance was notably high, not only between different strains but also among biological replicates. This high variability is attributed primarily to the inherent challenges associated with the cultivation of pneumococci under laboratory conditions. Consequently, significant differences between the serotypes could not be determined (Figure 3C).

## 3. Discussion

The prebiotic, antimicrobial and immune-modulatory activities of HMOs depend on their structure. More than 200 individual structures have been identified thus far, and the composition strongly varies between individual lactating women [10]. Anti-infective properties against certain pathogens have been reported for some HMOs. The most prominent active compounds in our HMO fraction were 2′-FL, 3′-FL, LDFT, LNT, LNnT, LNFP I–III, and LNDFH I/II, followed by 3′-SL and 6′-SL. These results were consistent with published compositional profiles of mature human milk [26,27]. We still detected traces of fatty acids and their derivatives in the HMO fraction. Although these compounds accounted for only a minor proportion of the total ion intensity compared with that of glycosidic species, their presence indicates that even multistep purification of human milk does not completely remove amphiphilic contaminants. This issue has been reported previously and often requires additional lipid-removal steps, such as Folch or C18 cleanup procedures [28]. However, given their very low abundance, these residual fatty acids are unlikely to significantly affect the antimicrobial activity attributed to the HMO fraction. Therefore, we chose not to apply further purification steps, as this could have led to an undesirable loss of HMOs during processing.

Most studies on HMOs address the gastrointestinal compartment and related pathogens (e.g., *Escherichia coli*, *Salmonella enterica*, *Helicobacter pylori*, Group B streptococci, norovirus and rotavirus), while some also address selected respiratory pathogens such as *Pseudomonas aeruginosa*, respiratory syncytial virus (RSV), and influenza virus [29]. In this study, we explored the antimicrobial effects of HMOs from pooled human milk on a range of *S. pneumoniae* isolates obtained from community-acquired pneumonia. To our knowledge, this is the first study addressing both vaccine-targeted serotypes and NESp isolates. By testing their impact on pneumococcal growth, we aimed to better understand the potential of HMOs as a complementary approach to vaccination in preventing pneumococcal respiratory infections, highlighting the beneficial effects of breastfeeding in providing natural protective factors against pneumococci in infants. We interpreted the growth curves and assessed CFU/mL changes on the basis of the HMO concentration and compared the effects to those associated with the typical lactose concentration.

We showed that HMOs possess potent and direct antimicrobial activity against *S. pneumoniae* independent of the serotype by inhibiting the growth and killing pneumococci within a concentration range that corresponds to the natural presence of HMOs in breast milk. These concentrations are reported to be highest in colostrum (the first form of milk after birth) at 20–25 mg/mL and decrease to 4–6 mg/mL after 6 months of lactation [29]. Approximately 10 g of HMOs are estimated to be consumed daily by breastfed infants [30]. These results underscore the potential of HMOs to serve as a natural defense mechanism to protect against and prevent pneumococcal colonization and infection of the nasopharynx and respiratory tract in breastfed infants, thereby complementing existing vaccination efforts and offering an additional layer of protection for high-risk populations.

The antipneumococcal activity observed in this study included serotypes that are less responsive to vaccines, such as serotype 3, as well as NESp isolates, which are not targeted by current serotype-based vaccines because of the absence of a polysaccharide capsule. Thus, the antipneumococcal effect is not directly dependent on capsular polysaccharides but rather involves other bacterial surface factors. Interestingly, compared with other serotypes, serotype 3 has a uniquely thick and mucoid capsule [31], a feature that has been associated with poor immunogenicity and reduced vaccine responsiveness. Despite this, serotype 3 was among the most susceptible to HMOs in our assays, suggesting that the interaction between HMOs and the bacterial surface is not solely restricted by capsule thickness. Instead, it is conceivable that HMOs may penetrate or interact with the hydrated polysaccharide matrix, possibly binding to surface-exposed adhesins or altering local osmotic conditions. In contrast, in other encapsulated serotypes, the capsule may exert a modest shielding effect, slightly reducing the efficacy of HMOs. However, this attenuation is unlikely to be clinically significant at the concentrations naturally present in human milk.

A possible explanation for the growth-inhibitory effect is interference with pneumococcal adhesins. As shown previously in vitro, the HMO fraction can inhibit the binding of pneumococci to nasopharyngeal epithelial cells. Sialylated and fucosylated HMOs may mask host receptors or bind to pneumococcal adhesins, thereby reducing colonization efficiency independent of maternal antibodies present in breast milk [32,33]. Earlier studies also demonstrated a reduction in the *S. pneumoniae* burden in a rabbit lung model following treatment with LNnT and its α2-6-sialylated derivative [34]. LNnT is structurally similar to the human epithelial receptor GlcNAcβ1-3Gal, which is recognized by several pathogens, including pneumococci. However, this adhesion-blocking mechanism alone cannot fully explain the bactericidal and lytic effects observed in our experiments.

A more plausible explanation for the bactericidal effect of HMOs may involve the dysregulated activation of pneumococcal glycosidases, particularly the neuraminidase NanA and the β-galactosidase BgaA. These surface-associated enzymes normally act on host glycoconjugates by sequentially removing terminal sialic acid and β-galactose residues [35]. This process exposes underlying GlcNAc-containing structures that serve both as adhesion receptors and as nutrient sources for *S. pneumoniae* [36]. Upon exposure to soluble HMOs, which are rich in sialylated and galactosylated motifs structurally resembling host glycans, similar enzymatic and transport pathways may be induced. However, many HMOs are only partially or not at all degradable by pneumococcal enzymes, leading to the accumulation of unprocessable sugar intermediates, redox imbalance, and eventual energy depletion. This metabolic stress can trigger autolysis through the activation of the autolysin LytA, a well-established mechanism of pneumococcal self-lysis under conditions of metabolic dysregulation [37]. Investigating this interaction at the molecular level would therefore be highly interesting. The degradation products of HMOs might represent valuable new compounds for combating pneumococcal infections and could be synthesized more easily than natural HMOs, of which only a few are currently available commercially.

We did not design our experiments to analyze pneumococcal biofilms; nevertheless, it is possible that the biofilms formed under the culture conditions used. Therefore, we cannot draw conclusions in this study regarding the effects of HMOs on pneumococcal biofilms. Nonetheless, HMOs have demonstrated antibiofilm activity against other pathogens and can modulate biofilms. In particular, numerous studies have investigated these effects in enteric pathogens, as reviewed elsewhere [38]. *S. pneumoniae* has not yet been examined in this context, highlighting the need for future pneumococcus-focused biofilm studies using defined HMO structures. Specifically, the antiadhesive properties of 2′-FL, 3′-FL, 3′-SL, and 6′-SL, as well as the biofilm-inhibitory effects of LNnT—previously demonstrated against group B streptococci—may also be effective against pneumococci [38].

*S. pneumoniae* has an exceptionally flexible genome characterized by high genetic variability and the ability to undergo horizontal gene transfer, particularly through natural transformation [39]. This genetic plasticity enables rapid adaptation to changing environmental conditions and selection pressures. The high recombination rate contributes to the emergence of new capsule types, allowing for pneumococci to evade immune recognition and, consequently, the protective effects of existing vaccines. Moreover, genetic exchange promotes the development of antibiotic resistance, complicating the treatment of pneumococcal infections. NESp has been shown to exhibit a higher transformation rate than its encapsulated counterparts do [40]. Additionally, 80–96% of isolated NESp strains are resistant to multiple antibiotics, including erythromycin, clindamycin, tetracycline, sulfamethoxazole-trimethoprim, and penicillin [41]. This provides NESp with a selective advantage while simultaneously making it a pneumococcal reservoir for antibiotic resistance genes. Although the benefits and drawbacks of pneumococcal vaccination currently remain in balance, vaccine-induced selection is likely to drive a continued increase in NESp-associated disease prevalence, highlighting the growing need for alternative therapeutic options.

Epidemiological studies have demonstrated that compared with nonbreastfed children, breastfed children experience a reduced incidence of respiratory infections, which is likely attributed to the protective properties of HMOs [42]. These findings highlight the potential of HMOs as natural therapeutic agents, not only for supporting a healthy gut microbiome during early life but also for preventing pneumococcal infections, particularly in high-risk populations. Future studies should investigate the in vivo efficacy of HMOs, their interactions with other bioactive milk components, and their potential therapeutic applications in clinical settings and in infectious foci such as blood, cerebrospinal fluid and pulmonary epithelial lining fluid. Moreover, longitudinal studies analyzing nasal swabs from children on different diets, such as natural milk or specific nutritional supplements, would provide more meaningful insights into the potential of HMOs as antipneumococcal agents. Future work should also dissect adhesion- versus enzyme-mediated mechanisms (e.g., neuraminidase or exoglycosidase activity) using *nanA* and *bgaA* mutants and purified HMO species such as 2′-FL, 3′-SL, 6′-SL, and LNnT, as well as employ dedicated pneumococcal biofilm models to elucidate structure–activity relationships.

## 4. Materials and Methods

### 4.1. Bacterial Strains

Clinical *S. pneumoniae* isolates were provided anonymously by the Reference Laboratory for Streptococci (Aachen, Germany). The isolates were serotyped using Neufeld’s Quellung reaction as described previously [43] and stored at −80 °C in Cryosystem Protect Beads Tubes (Sarstedt AG & Co. KG, Nümbrecht, Germany). The strains were freshly streaked from cryopreservations onto blood agar plates (Becton Dickinson GmbH, Heidelberg, Germany) and incubated overnight at 35 °C with 5% CO_2_. Fresh bacterial suspensions were prepared from several single colonies in Todd-Hewitt (TH) broth (Carl Roth GmbH + Co. KG, Karlsruhe, Germany) and adjusted to an optical density of 0.1 at 600 nm using a Multiskan™ GO photometer (Thermo Fisher Scientific Inc., Waltham, MA, USA).

### 4.2. HMOs and Lactose

Human milk was collected from healthy volunteers in a study approved by the ethics committee of the Medical University of Warsaw in October 2018 under the registry number AKBE/180/2018 [20]. All participants provided written informed consent prior to inclusion. The Ethics Committee of Jena University Hospital approved the anonymized collaborative use of the material (Jena, Germany; registry number 2019-1360-Material). The donors were between three and six months postpartum at the time of sampling and provided 200–250 mL aliquots that were immediately frozen after collection.

In total, 2.1 L of human milk was obtained by pooling nine thawed aliquots collected from nine different healthy donors. The pooled milk was centrifuged twice at 3000× *g* for 20 min at 4 °C in a 5804R table centrifuge (Eppendorf SE, Hamburg, Germany) to remove the fat layer, and the resulting skim milk was collected. The proteins were precipitated by adding cold 96% ethanol at a 1:1 (*v*/*v*) ratio to the skim milk, followed by overnight incubation at 4 °C. The mixture was subsequently centrifuged at 3000× *g* for 30 min at 4 °C, after which the supernatant containing the carbohydrates was recovered. Ethanol was removed from the supernatant using a rotary evaporator (Laborota 4000, Heidolph Scientific Products GmbH, Schwabach, Germany) at 78 °C, and the dried residue was reconstituted in deionized water. The resulting solution was freeze-dried using a VaCo 2 laboratory freeze dryer (Zirbus Technology GmbH, Bad Grund, Germany) to obtain a powder enriched in milk oligosaccharides. The final product was stored at 4 °C until further analysis.

Lactose (Carl Roth GmbH + Co. KG) was used as a control, as previous analyses revealed that the oligosaccharide fraction contains approximately 80–90% lactose after extraction [44]. We intentionally did not enzymatically remove lactose from the HMO fraction, since hydrolysis would generate glucose and galactose—the preferred carbon sources for *S. pneumoniae* [36]—potentially confounding bacterial growth and metabolic responses.

The HMOs and lactose were freshly prepared as 20 mg/mL and 200 mg/mL stock solutions, respectively, in TH broth before use.

### 4.3. NMR Spectroscopy

For nuclear magnetic resonance (NMR) spectroscopy, 10.4 mg of the HMO fraction was dissolved in 0.75 mL of the deuterated form of dimethyl sulfoxide (DMSO-d_6_) (99.9% D, Merck, Germany). The spectra were recorded via a Bruker Avance III 600 spectrometer (Bruker AXS Inc., Madison, WI, USA) (600.15 MHz for ^1^H) equipped with a 5 mm QCI CP^2^ cryoprobe. The relaxation delay (D_1_) was set to 10 s to ensure quantitative peak integration.

### 4.4. LC–MS Analysis of HMOs

To analyze and quantify the sugar content of the HMO fraction, ultrahigh-performance liquid chromatography (UHPLC) coupled with high-resolution mass spectrometry (HRMS) was performed. Chromatographic separation of HMOs was performed via a Thermo UltiMate HPG-3400 RS binary pump connected to a WPS-3000 autosampler maintained at 10 °C, and equipped with a 25 µL syringe and a 100 µL sample loop, an autosampler and a UV detector, an a Hypercarb porous graphitic carbon (PGC) column (3 µm, 100 × 2.1 mm) (all Thermo Fischer Scientific). The column temperature was kept constant at 25 °C using a TCC-3200 compartment (Dionex Softron, Germering, Germany). The mobile phases consisted of (A) 5 mM ammonium acetate (pH 9.6) + 2% acetonitrile and (B) 5 mM ammonium acetate (pH 9.6) + 80% acetonitrile. The gradient was applied as follows: 0 min—90% B; 0.2 min—90% B; 11 min—70% B; 13 min—0% B; 15 min—0% B; 15.1 min—90% B; and 17 min—90% B at a flow rate of 0.22 mL min^−1^. The injection volume was 2 µL, and the total run time was 17 min.

Mass spectrometric analysis of the fractions previously separated by a PGC column was carried out using a Q Exactive Plus Orbitrap mass spectrometer (Thermo Scientific, Waltham, MA, USA) equipped with an electrospray ionization (ESI) source operating in both positive and negative ion modes. The scan range was *m*/*z* 150–2000, with a mass resolution of 140,000 FWHM at *m*/*z* 200. Calibration was performed daily with the manufacturer’s standard calibration mix.

### 4.5. LC–MS Analysis of the General Compound Composition of the HMO Fraction

To analyze the general composition of the HMO fraction, UHPLC–HRMS was performed using the same system as mentioned above. A flow rate of 0.4 mL min^−1^ was applied for all runs. Chromatographic separations were carried out using two different column systems, each operated under specific solvent conditions and gradients: For reversed-phase separation, an Accucore^®^ C18 column (100 × 2.1 mm, 2.6 µm) (Thermo Fisher Scientific) was used. The mobile phases consisted of (A) water with 2% acetonitrile and 0.1% formic acid and (B) pure acetonitrile. The gradient started at 0% B and was held for 0.2 min, followed by a linear increase to 100% B over 8 min. This condition was maintained until 11 min before the initial composition (0% B) was returned and the sample was equilibrated for 1 min, resulting in a total run time of 12 min. To separate the hydrophilic interactions, a SeQuant ZIC-HILIC column (150 × 2.1 mm, 5 µm) fitted with a ZIC-HILIC guard column (20 × 2.1 mm, 5 µm) (Merck KGaA, Darmstadt, Germany) was employed. The mobile phases were (A) water containing 0.1% formic acid and (B) acetonitrile with 0.1% formic acid. The gradient began at 100% B, held for 0.2 min, then decreased linearly to 0% B over 8 min, was maintained until 11 min, and then returned to the initial 100% B for re-equilibration. The total runtime was 12 min.

Mass spectra were acquired via a Q Exactive Plus Orbitrap mass spectrometer (Thermo Fisher Scientific, Waltham, MA, USA) equipped with a heated ESI source. To minimize source contamination, the column flow was directed to the mass spectrometer between 0.5 and 11.5 min and diverted to waste outside this time window. Data were acquired in both positive and negative full-scan modes with the following parameters: *m*/*z* 100–1500, resolution 70,000 (at *m*/*z* 200), AGC target 3 × 10^6^, and maximum injection time 200 ms. The general source settings were as follows: sheath gas flow rate 60, auxiliary gas 20, sweep gas 5, spray voltage 4.0 kV in positive mode (2.5 kV in negative mode), capillary temperature 320 °C, S-lens radio frequency level 90, and auxiliary gas heater temperature 400 °C. The acquisition time frame was 0.5–11.5 min for all runs.

Data analysis was performed using Thermo Compound Discoverer 3.2.0.421, employing a metabolomics workflow with database searches in ChemSpider, mzCloud, and mzVault for molecular formula annotation and compound identification.

### 4.6. Growth Curves

The growth experiments were conducted in 96-well plates (Greiner Bio-One International GmbH, Frickenhausen, Germany). Lactose and HMO stock solutions were diluted in TH broth to twice the final concentrations. In each well, 150 µL of the adjusted bacterial suspension and 150 µL of the diluted lactose or HMOs were mixed, and the growth in a 5% CO_2_ atmosphere was recorded using an Infinite PRO 200 microplate reader (Tecan Group AG, Männedorf, Switzerland) every 15 min at 600 nm for 16 h at 35 °C. The OD_600_ was measured 5 times per well, and the data were averaged. The experiments were performed in duplicate for every strain and condition. The following concentrations (in mg/mL) were tested: (A) HMOs at 1, 1.5, 2, 2.5, 5, and 10; and (B) lactose 0.8, 1.2, 1.6, 2, 4, and 8.

### 4.7. Visualization and Mathematics

The blank-subtracted growth curves are presented in the Supplementary Materials (Figures S2–S7), with average values ranging between 0.1004 (±0.0002) and 0.1075 (±0.0018). Thus, we decided to use the average of the first two measured values (starting point and after 15 min) for normalization so that the OD_600_ values were set to zero at the starting point.

**Figure 4:**
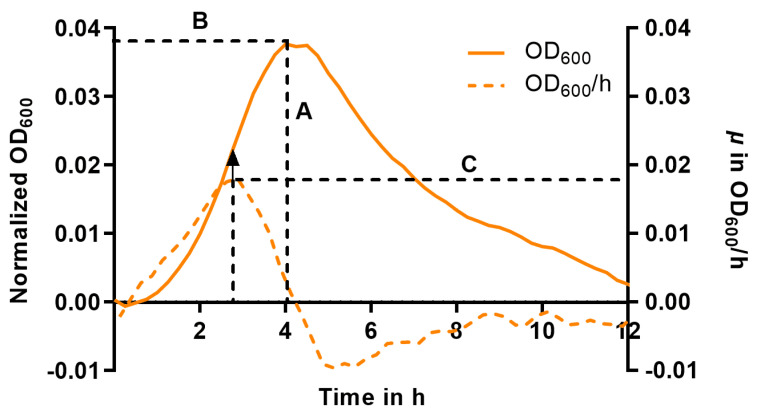
Schematic representation of the growth parameters (A to C) used in this study to evaluate the effects of HMOs on pneumococcal growth demonstrated on an exemplary curve after baseline normalization (solid line, left *y*-axis) and its first corresponding derivative (dashed line, right *y*-axis). A = maximum OD (OD_MAX_), B = t at OD_MAX_ (t_MAX_), C = maximum growth rate (µ_MAX_) at the inflection point. The arrow indicates the turning point of the growth curve that corresponds to the maximum of the derivative function representing the µ_MAX_ (C).

To describe the effect of the treatment, we determined the maximum of the curve (Figure 4, A), which is equivalent to the maximum achievable bacterial number (OD_MAX_), and the corresponding time to achieve this maximum (t_MAX_) (Figure 4, B). In cases where the curves exhibited a consistently decreasing trend from the beginning, OD_MAX_ and t_MAX_ were set to zero, as no growth occurred. Using the first derivative of the growth curve, we determined the inflection point of the logarithmic phase (maximum at the respective time) corresponding to the maximum growth rate µ_MAX_ [h^−1^] (Figure 4, C).

### 4.8. Counting of Viable Bacteria

Viable bacteria were assessed on TH agar plates. The adjusted bacterial suspension (OD_600_ of 0.1) and the HMO or lactose solutions were prepared as described above. The cultures were prepared in sterile 15 mL conical polypropylene tubes (Greiner Bio-One International GmbH) by mixing equal volumes of the bacterial suspension and twice-concentrated HMOs or lactose solutions to a final volume of 3 mL each. After incubation for 6 h at 35 °C under orbital rotation (115 rpm), the bacterial cultures were serially diluted (between 10^0^ and 10^−4^), and 10 µL was added to TH agar in triplicate for each dilution and incubated overnight at 35 °C in a 5% CO_2_ atmosphere. Thereafter, the number of colony-forming units (CFU/mL) was determined from countable droplets (between 1 and 50 CFU per spot). The concentration at which no colonies could be determined was estimated as the killing concentration. The experiments were performed in biological triplicates.

### 4.9. Statistics

The parameters were calculated for each condition and for each replicate and were used for statistical assessment of the effects by two-way ANOVA followed by Sidak’s multiple comparison test in GraphPad Prism 10 (GraphPad Software, Inc., Boston, MA, USA).

## Data Availability

All the data supporting the findings of this study are presented within the article and Supplementary Materials. Raw data are available from the corresponding author upon reasonable request.

## Supplementary Materials

The following supporting information can be downloaded at https://www.mdpi.com/article/10.3390/ijms262110781/s1.

## Abbreviations

CFU	Colony-forming unit
DFLNH	Difucosyllacto-*N*-hexaose
DFpLNH	Difucosyl-para-lacto-*N*-hexaose
DMSO-d_6_	Deuterated form of dimethyl sulfoxide
ESI	Electrospray ionization
2′FL	2′-Fucosyllactose
3′FL	3-Fucosyllactose
Gal	Galactose
Glc	Glucose
GlcNAc	*N*-acetyl-glucosamine
HMOs	Human milk oligosaccharides
IFLNH	Isomerfucosyllacto-*N*-hexaose
IPD	Invasive pneumococcal disease
LDFT	Lacto-di-fructotetraose
LC	Liquid chromatography
LNDFH	Lacto-*N*-di-fucohexaose
LNFP	Lacto-*N*-fucopentaose
LNH	Lacto-*N*-hexaose
LNnH	Lacto-*N*-neohexaose
LNnT	Lacto-*N*-neotetraose
LNT	Lacto-*N*-tetraose
LST	Lacto-*N*-sialyltetraose
MFLNH	Monofucosyllacto-*N*-hexaose
MFpLNH	Monofucosyl-para-lacto-*N*-hexaose
µ_MAX_	Maximum growth rate
NMR	Nuclear magnetic resonance
MS	Mass spectrometry
OD	Optical density
OD_MAX_	Maximum achievable OD
t_MAX_	Time to achieve OD_MAX_
NESp	Nonencapsulated *S. pneumoniae*
PCV	Pneumococcal conjugate vaccine
PGC	Porous graphitic carbon
3′S3FL	3′-Sialyl-3-fucosyllactose
3′SL	3′-Sialyllactose
6′SL	6′-Sialyllactose
ST	Serotype
TFLNH	Trifucosyllacto-N-hexaose
TH	Todd-Hewitt
UHPLC	Ultrahigh-performance liquid chromatography
