# Supplementary material for: Anti-Pneumococcal Properties of the Native Human Milk Oligosaccharide Fraction: A Concentration-Dependent Study
Source: Int J Mol Sci. 2025 Nov 6;26(21):10781. doi: 10.3390/ijms262110781 (PMC12609556; doi:10.3390/ijms262110781)
Supplement: Supplementary file 1 [file ijms-26-10781-s001.zip › ijms-3937989-supplementary.pdf]

Supplementary Material

Anti-pneumococcal Properties of the Native Human Milk Oligosaccharide Fraction: A Concentration-Dependent Study

Authors

Oliwia Makarewicz [1]*, Tinatini Tchatchiashvili [1], Lisa Jasef [1], Mark P.G. van der Linden [2], Sylwia Jarzynka [3], Kamila Strom [3], Nico Ueberschaar [4], Maciej Mazur [5], Gabriela Oledzka [3] and Mathias. W. Pletz [1]

[1] Institute of Infectious Diseases and Infection Control, Jena University Hospital, Friedrich Schiller University Jena, Am Klinikum 1, 07747 Jena, Germany; tinatini.tchatchiashvili@med.uni-jena.de (T.T.); lisa.jasef@med.uni-jena.de (L.J.); mathias.pletz@med.uni-jena.de (M.W.P.)
[2] Uniklinik RWTH Aachen, Institute of Medical Microbiology, Referenzlabor für Streptokokken, Pauwelstraße 30, 52074 Aachen, Germany; mlinden@ukaachen.de
[3] Department of Medical Biology, Medical University of Warsaw, Litewska 14/16, 00-575 Warsaw, Poland; sylwia.jarzynka@wum.edu.pl (S.J.); kamila.strom@wum.edu.pl (K.S.); gabriela.oledzka@wum.edu.pl (G.O.)
[4] Mass Spectrometry Platform, Friedrich Schiller University Jena, Humboldtstraße 8, 07743 Jena, Germany
[5] Faculty of Chemistry, University of Warsaw, Pasteura 1, 02-093 Warsaw, Poland; mmazur@chem.uw.edu.pl
* Correspondence: oliwia.makarewicz@med.uni-jena.de; Fax: +49-36419324227

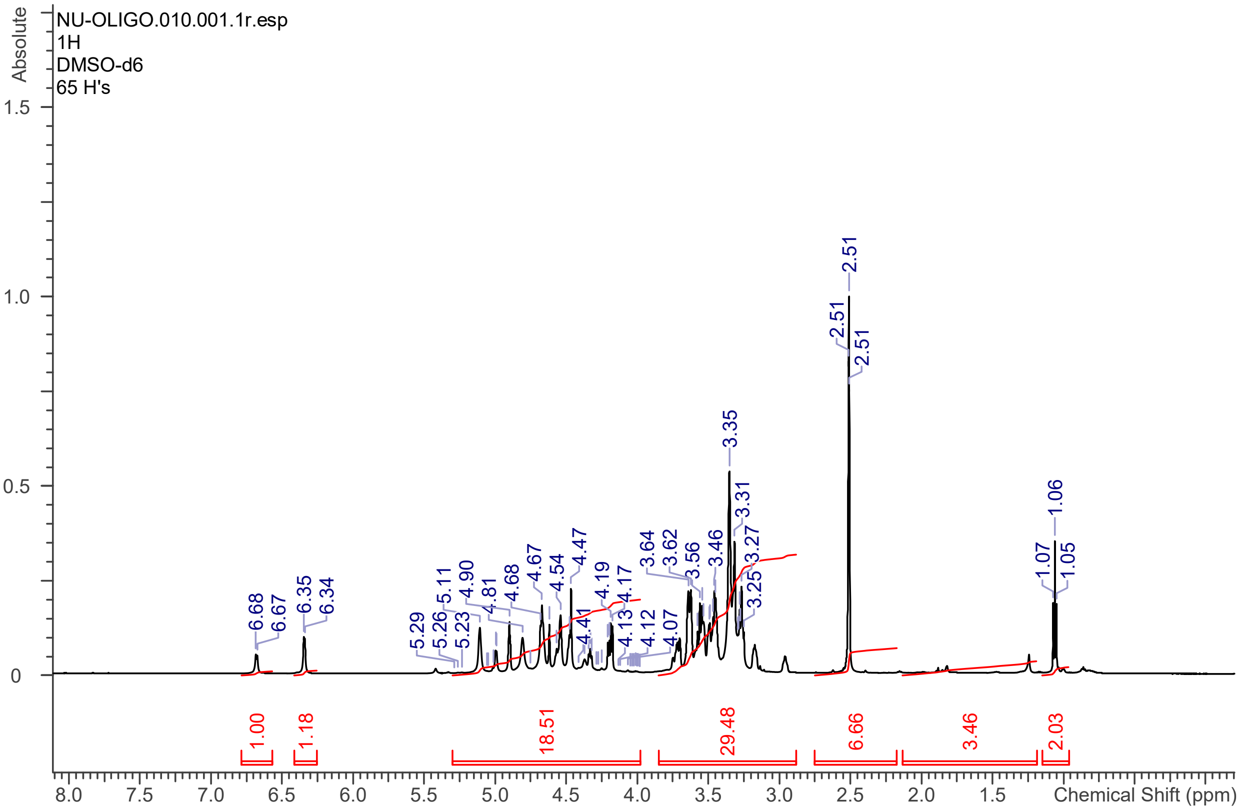

Figure S1: 600 MHz proton NMR-spectrum of HMO fraction in DMSO-D6. (2.51 ppm, solvent residual peak)

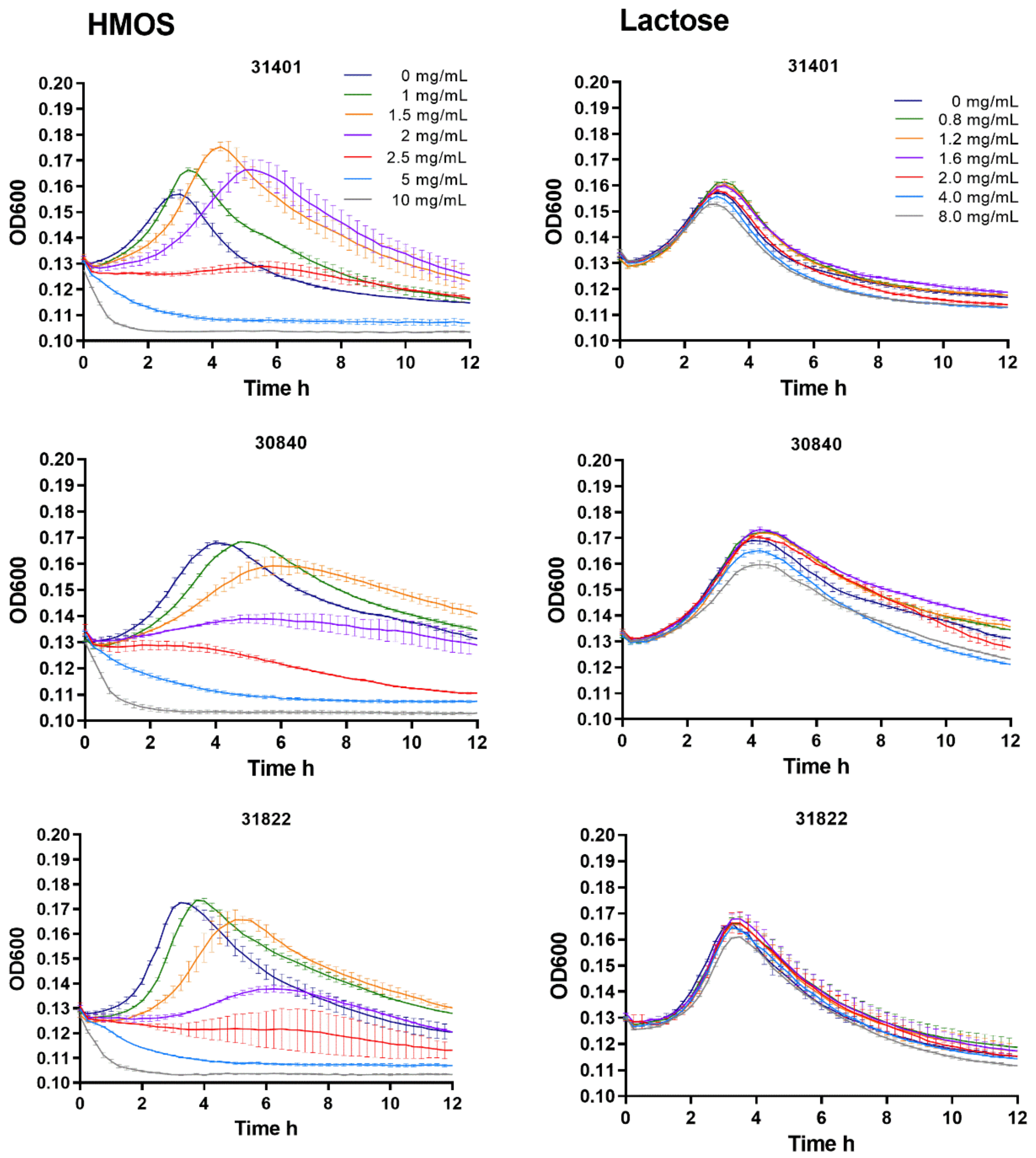

Figure S2: Growth curves of serotype 3 *Streptococcus pneumoniae* isolates grown under different concentrations of HMOs or lactose. Blank was subtracted from all growth curves. Isolates are indicated above each diagram.

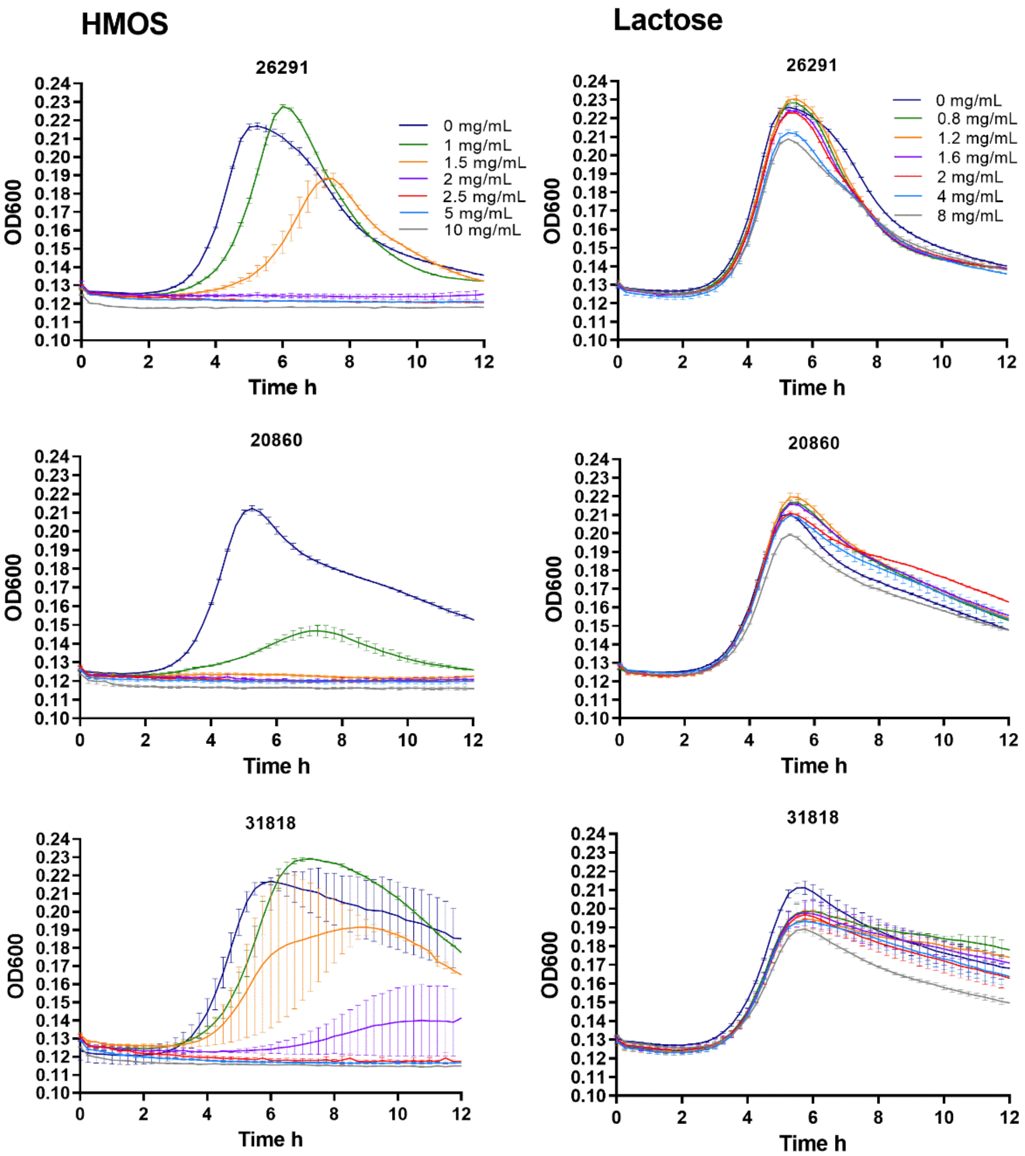

Figure S3: Growth curves of serotype 6B *Streptococcus pneumoniae* isolates grown under different concentrations of HMOs or lactose. Blank was subtracted from all growth curves. Isolates are indicated above each diagram.

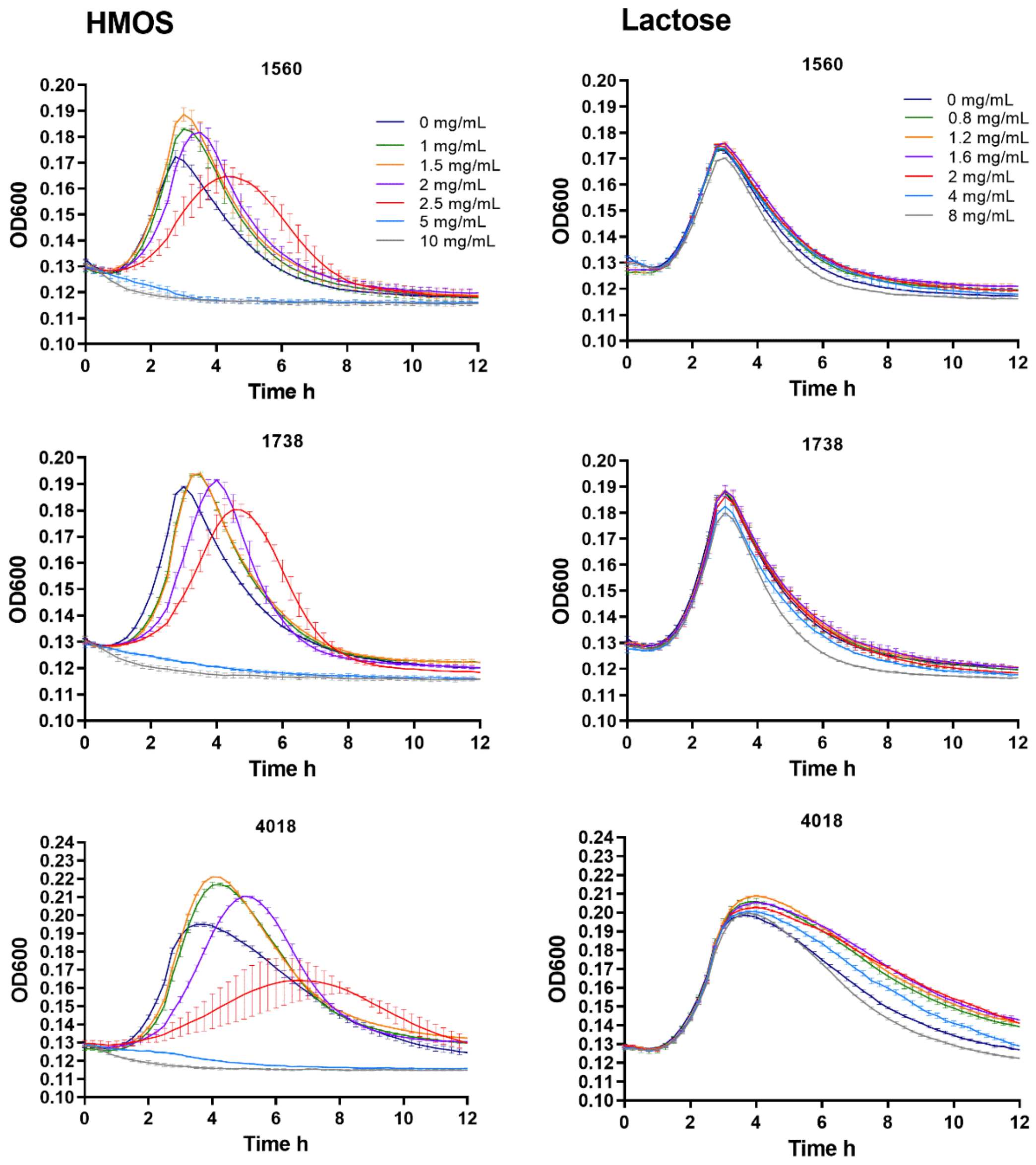

Figure S4: Growth curves of serotype 14 *Streptococcus pneumoniae* isolates grown under different concentrations of HMOs or lactose. Blank was subtracted from all growth curves. Isolates are indicated above each diagram.

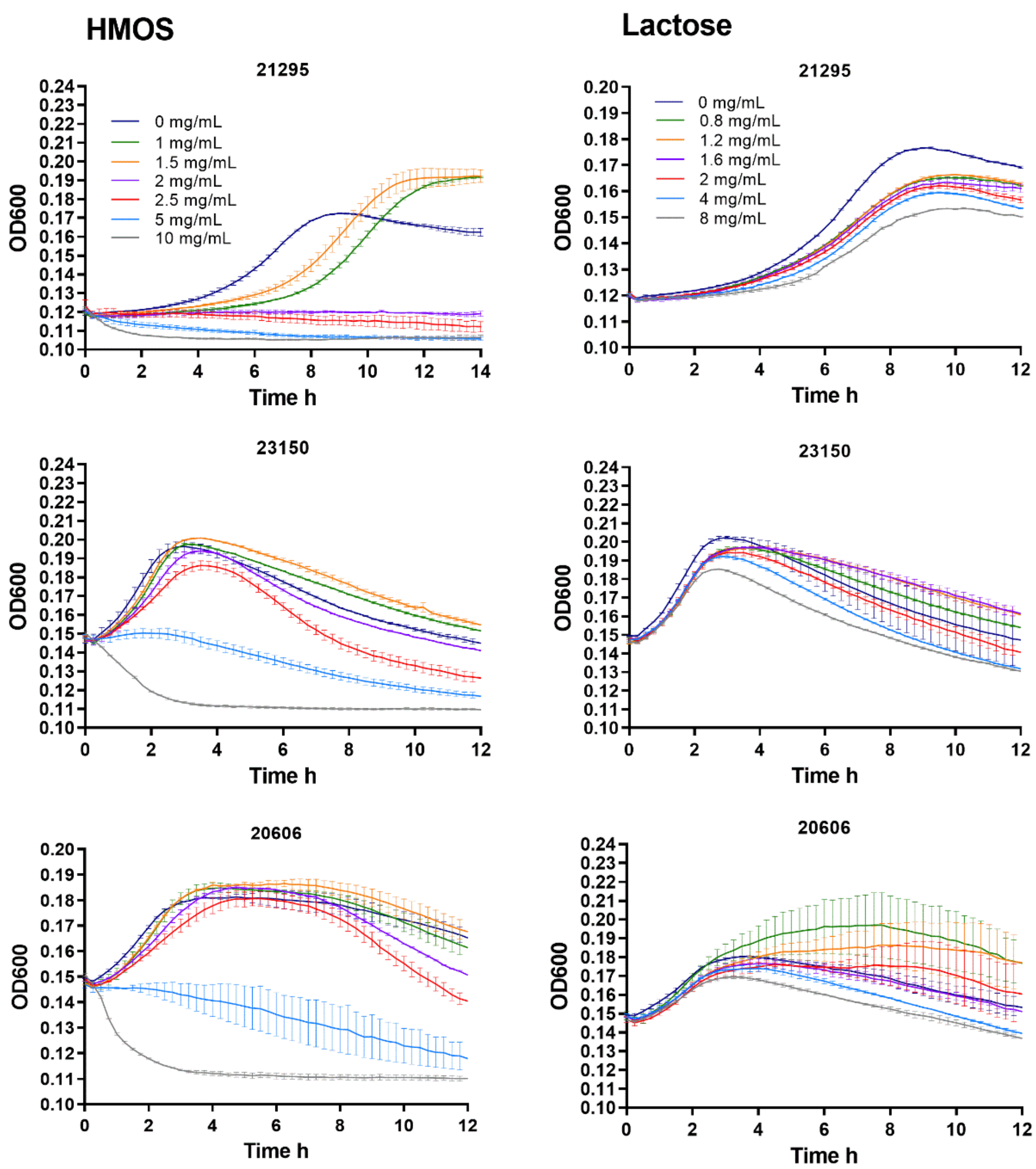

Figure S5 Growth curves of serotype 19A *Streptococcus pneumoniae* isolates grown under different concentrations of HMOs or lactose. Blank was subtracted from all growth curves. Isolates are indicated above each diagram.

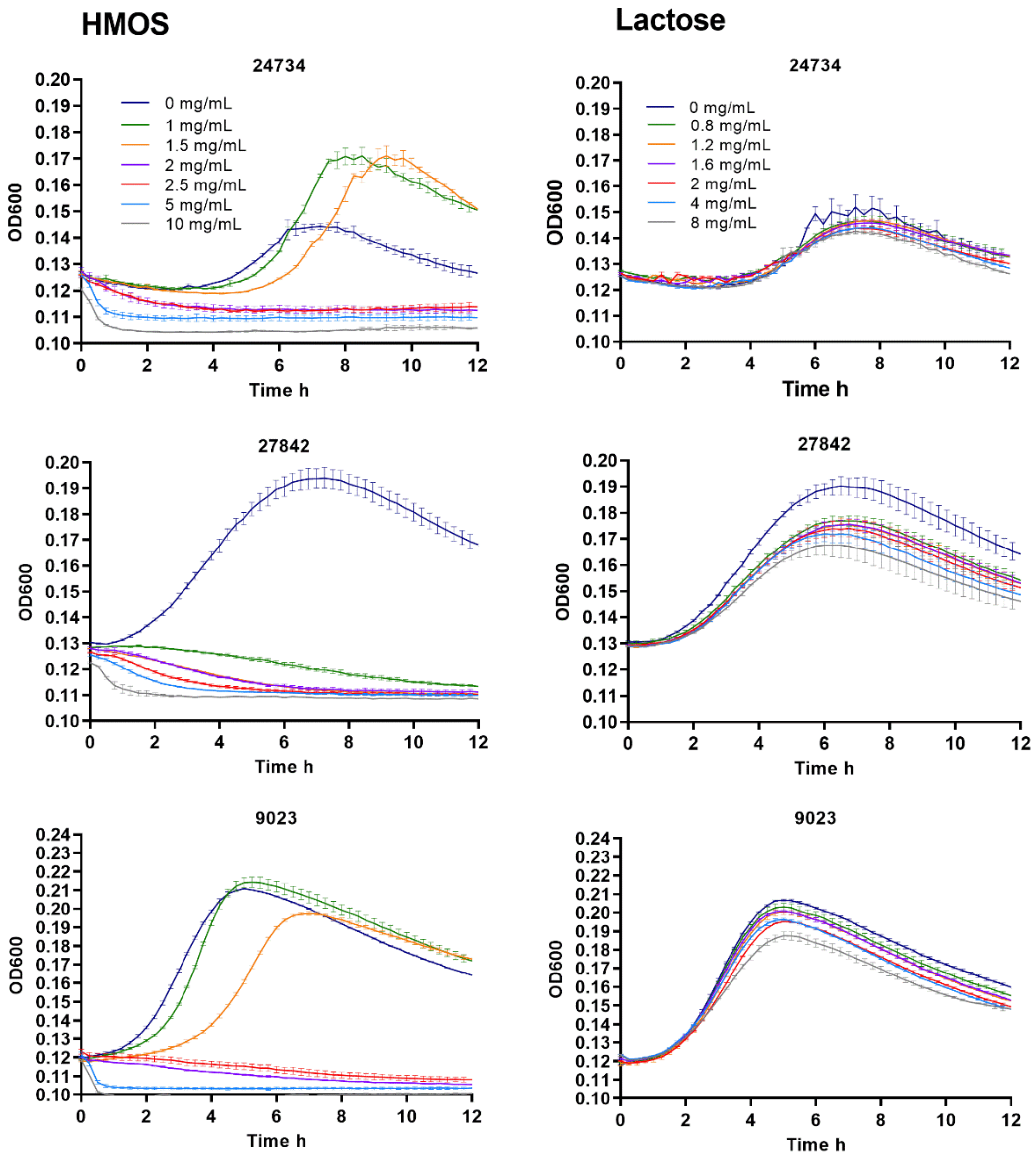

Figure S6: Growth curves of serotype non-encapsulated *Streptococcus pneumoniae* isolates grown under different concentrations of HMOs or lactose. Blank was subtracted from all growth curves. Isolates are indicated above each diagram.

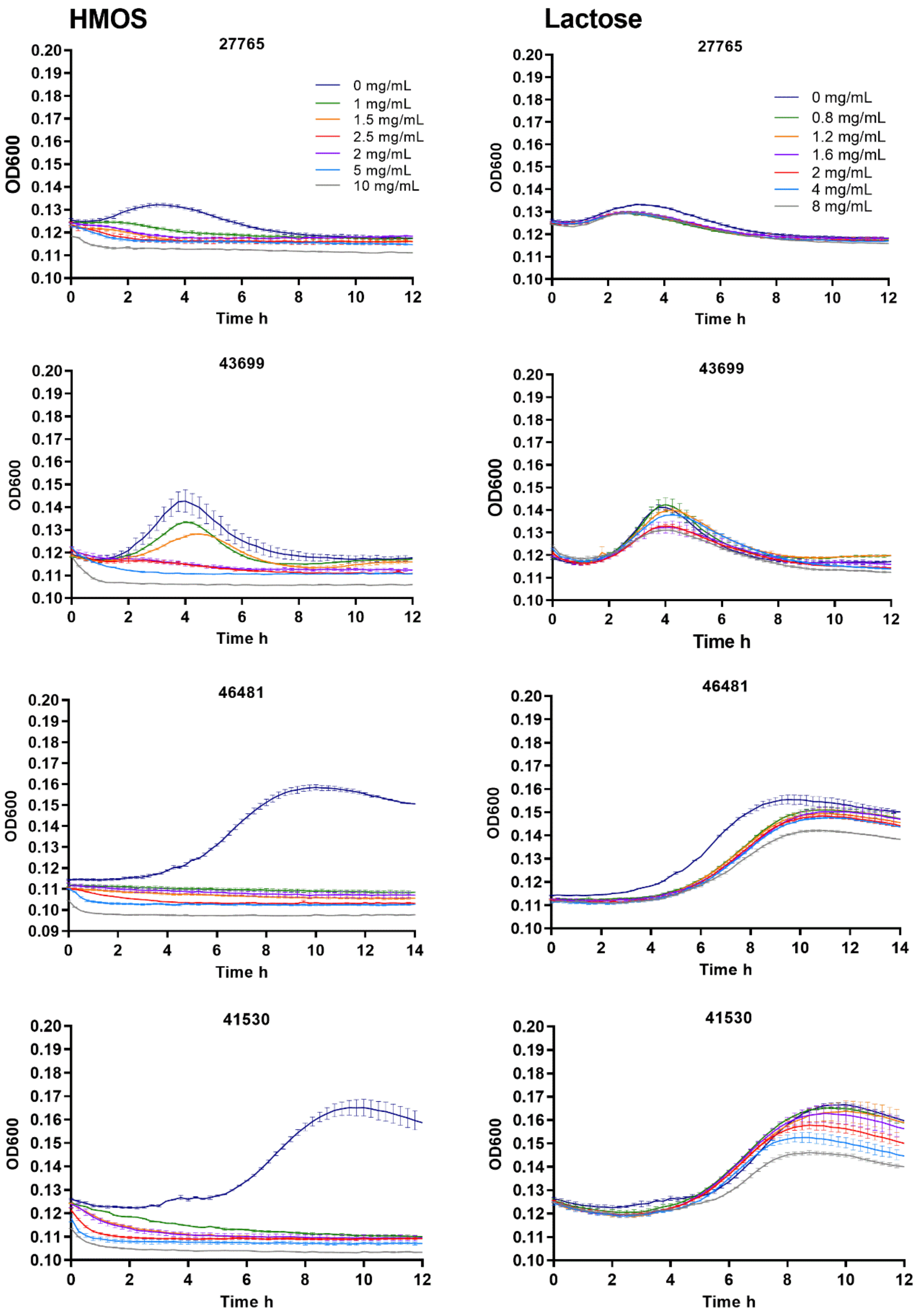

Figure S7: Further Growth curves of serotype non-encapsulated *Streptococcus pneumoniae* isolates grown under different concentrations of HMOs or lactose. Blank was subtracted from all growth curves. Isolates are indicated above each diagram.

Table S1: Relative abundance of saccharide types in the analysed HMO fraction, calculated on the basis of the integrated EIC peak areas of the total HMOs fractionated.

Saccharide type	Integrated EIC intensity ($[M+K]^+$)	Relative abundance (%)
Monosaccharide	4.650×10^4	0.14 %
Disaccharide	2.59×10^7	77.71 %
Trisaccharide	7.05×10^6	21.15 %
Tetrasaccharide	1.31×10^5	0.39 %
Pentasaccharide	1.66×10^5	0.5 %
Hexasaccharide	3.45×10^4	0.1 %

Table S2: Compound annotation of the HMO fraction using a C18 reversed phase chromatography

Compound / Fragment assignment	Formula	Calc. MW	m/z	Reference ion	ΔMass [ppm]	RT [min]	Area (Max.)
**1-α-D-Galactosyl-myo-inositol**	C12H22O11	342.11583	365.10489	[M+Na]+	−1.11	1.075	5.59E+07
**Oleic acid**	C18H34O2	282.25445	281.24716	[M−H]−	−3.0	9.322	3.67E+07
**Fragment of amino acid / peptide derivative**	C11H16N4O2	236.12703	254.16086	[M+NH4]+	−1.23	1.139	2.22E+07
**4-Methyl-7,11-heptadecadienal**	C18H32O	264.24453	282.27835	[M+NH4]+	−2.97	8.739	2.05E+07
**1-Palmitoyl-glycerophosphocholine**	C24H50NO7P	495.33132	496.33856	[M+H]+	−2.36	6.757	1.89E+07
**Fragment of fatty acid sulfonate**	C17H28O3S	312.175	311.16772	[M−H]−	−2.93	9.322	1.70E+07
**Fragment of sulfated fatty acid**	C21H40O2S	356.27384	339.27058	[M+H−H2O]+	−2.98	7.689	1.52E+07
**Arachidonic acid (FA 20:4)**	C20H32O2	304.2393	303.23203	[M−H]−	−3.05	8.54	1.47E+07
**Peptide fragment (amino acid rich)**	C15H26N6O3	338.20598	339.21326	[M+H]+	−1.95	6.927	1.39E+07
**Diethylene glycol n-butyl ether**	C8H18O3	162.12545	185.11467	[M+Na]+	−0.92	3.63	1.39E+07
**Etifoxine**	C17H17ClN2O	300.102	299.09473	[M−H]−	−3.13	7.563	1.38E+07
**Fragment of polyamine / guanidine derivative**	C14H28N6	280.23875	279.23148	[M−H]−	+4.31	8.712	1.26E+07
**2,3,8,9-Tetrahydrocineromycin B**	C17H30O4	298.21358	299.22086	[M+H]+	−2.78	6.931	1.25E+07
**4-Dodecylbenzenesulfonic acid**	C18H30O3S	326.19115	325.18387	[M−H]−	−1.29	9.323	1.20E+07
**N-Acetyl-3,5,11,18-tetrahydroxy-octadecyl-2-amine**	C20H41NO5	375.29752	376.3048	[M+H]+	−2.54	7.161	1.09E+07
**Fragment of phosphatidylethanolamine headgroup**	C15H32N2O5P2	382.18056	381.17328	[M−H]−	+5.0	9.322	1.05E+07
**Butyryl-L-carnitine**	C11H21NO4	231.14656	232.15384	[M+H]+	−2.14	2.375	1.04E+07
**Docosahexaenoic acid (DHA, FA 22:6)**	C22H32O2	328.23991	327.23264	[M−H]−	−0.97	8.37	1.04E+07
**1-Linoleoyl-glycerophosphocholine**	C26H50NO7P	519.33158	520.33887	[M+H]+	−1.75	6.392	1.02E+07
**N-Acetyl-2,4,10,17-tetrahydroxy-heptadecylamine**	C19H39NO5	361.28184	362.28912	[M+H]+	−2.72	6.929	8.35E+06
**Fragment of chlorinated alkylamide compound**	C16H23ClN4O2	338.14973	339.15701	[M+H]+	−3.62	7.57	8.03E+06
**(R)-10-Hydroxystearic acid**	C18H36O3	300.26557	299.25829	[M−H]−	−2.91	7.636	7.72E+06
**Pantothenic acid (Vitamin B5)**	C9H17NO5	219.11036	220.11752	[M+H]+	−1.4	2.283	7.53E+06
**Fragment of nucleotide-sugar derivative**	C25H39N5O12	601.25761	602.26471	[M+H]+	−3.17	2.305	7.51E+06
**Fragment of glycerophospholipid**	C27H47N3O8	541.33473	540.32745	[M−H]−	−2.93	6.748	6.45E+06
**Duplicate of 4-Methyl-7,11-heptadecadienal**	C18H32O	264.24465	282.27847	[M+NH4]+	−2.52	8.935	6.38E+06
**Fragment of phosphatidylcholine**	C27H48N5O3P	521.34745	522.35474	[M+H]+	−3.88	6.92	5.75E+06
**Fragment of glycerophosphoethanolamine**	C26H43N5O4	489.32903	490.33631	[M+H]+	−5.05	7.287	5.56E+06
**3,6,9,12-Tetraoxa-docosan-1-ol**	C18H38O5	334.27104	376.30486	[M+ACN+H]+	−2.65	6.948	5.40E+06
**O-Propanoyl-carnitine**	C10H19NO4	217.131	218.13828	[M+H]+	−1.89	0.996	5.39E+06
**Vinyl-cyclohexene**	C8H12	108.0938	109.10108	[M+H]+	−0.97	3.278	5.38E+06
**Palmitic acid (FA 16:0)**	C16H32O2	256.23999	255.23274	[M−H]−	−0.92	9.414	5.11E+06

Table S3: Compound annotation of the HMO fraction using HILIC chromatography

Compound / Fragment assignment	Formula	Calc. MW	m/z	Reference ion	ΔMass [ppm]	RT [min]	Area (Max.)
**1-α-D-Galactosyl-myo-inositol**	C12H22O11	342.1152	387.11356	[M+FA−H]−	−2.95	4.967	1.78E+09
**Oligosaccharide fragment (HMO-related)**	C13H24O13	388.12102	387.11374	[M−H]−	−1.74	4.881	6.46E+08
**Choline**	—	103.09919	104.10647	[M+H]+	—	5.155	6.38E+08
**Fragment of glycerophosphocholine**	C24H29N2O7P	488.17134	533.16827	[M+FA−H]−	0.21	5.056	5.35E+08
**Fragment of phosphatidylethanolamine**	C18H22N3O3P	359.14041	360.14769	[M+H]+	1.5	4.938	5.26E+08
**Benserazide**	C10H15N3O5	257.10146	258.10874	[M+H]+	1.12	5.442	4.88E+08
**Small metabolite fragment**	—	161.1043	162.11158	[M+H]+	—	5.09	4.72E+08
**Fragment of sulfated aromatic compound**	C16H20O5S	324.10394	325.11116	[M+H]+	2.46	4.957	4.60E+08
**Inorganic background ion**	—	198.81487	197.80759	[M−H]−	—	4.139	4.59E+08
**Inorganic background ion**	—	196.81813	195.81085	[M−H]−	—	4.138	3.73E+08
**N-Lauryldiethanolamine**	C16H35NO2	273.26602	274.2733	[M+H]+	−2.78	3.084	3.71E+08
**Fragment of CDP-glycerol lipid**	C30H47NO14P2	707.2461	706.2403	[M−H]−	−1.52	5.318	2.87E+08
**Fragment of nucleotide-lipid intermediate**	C37H47N6O14P	830.28749	829.28094	[M−H]−	−1.56	5.005	2.18E+08
**Fragment of phosphorylated compound**	C3HN2O5P	175.96307	174.95579	[M−H]−	4.31	8.451	2.03E+08
**Fragment of glycoprotein residue**	C20H33NO14	511.18797	512.19525	[M+H]+	−4.17	5.44	1.99E+08
**2,3-Dimethylmaleic acid**	C6H8O4	144.0416	145.04877	[M+H]+	−4.59	4.964	1.64E+08
**Creatinine**	C4H7N3O	113.05842	114.0657	[M+H]+	−4.37	5.23	1.61E+08
**Fragment of nucleotide derivative**	C10H19N7O8	365.13014	366.13742	[M+H]+	1.74	5.318	1.61E+08
**D-(−)-Erythrose / L-Threose**	C4H8O4	120.04186	179.05569	[M−H+HAc]−	−3.35	4.97	1.54E+08
**Fragment of phosphatidylinositol**	C27H41NO11P2	617.21411	618.22064	[M+H]+	−2.23	5.391	1.43E+08
**Fragment of diphosphatidylglycerol (cardiolipin)**	C34H53NO16P2	793.28402	852.29785	[M−H+HAc]−	0.08	5.444	1.41E+08
**2,5-Dioxopiperazine**	C4H6N2O2	114.0424	132.07622	[M+NH4]+	−4.6	5.111	1.38E+08
**Fragment of phosphorylated sugar derivative**	C16H24O7P2S	422.07243	421.06516	[M−H]−	1.5	4.83	1.35E+08
**Fragment of nucleotide sugar conjugate**	C32H53N7O16P2	853.30158	854.30896	[M+H]+	−0.96	5.439	1.29E+08
**Fragment of ribosomal RNA base analog**	C44H55N8O17P	998.34153	997.33508	[M−H]−	−0.75	5.182	1.12E+08
**Fragment of polyphosphorylated nucleoside**	C16H32N9O12P	573.18919	632.20306	[M−H+HAc]−	−2.81	5.095	1.09E+08
**Fragment of nucleoside polyphosphate**	C40H50N7O15P	899.31013	898.30286	[M−H]−	−0.13	5.442	1.05E+08
**Metal adduct artifact**	—	123.89333	160.84193	[M−2H+K]−	—	4.12	1.05E+08
**Fragment of nucleotide sugar phosphate**	C19H30N5O12P	551.16542	550.15814	[M−H]−	4.65	5.079	1.04E+08
**Fragment of glycerophosphate**	C21H31NO7P2	471.15638	472.16345	[M+H]+	−2.53	5.351	1.02E+08
**Small metabolite fragment**	—	126.03104	127.03832	[M+H]+	—	4.941	1.01E+08
**Acetyl-L-carnitine**	C9H17NO4	203.11536	204.12263	[M+H]+	−1.96	4.221	8.96E+07
**Fragment of phosphatidylglycerol (PG)**	C14H24N2O8P2	410.					
